# Seeing radiation: Applications of an imaging survey meter in radiation oncology

**DOI:** 10.1002/acm2.70801

**Published:** 2026-09-23

**Authors:** Patrick N. McDermott, Robert C. Halford, Brayden Halvorson, Ian Lake, Jian Liang, Akin Ogretici, Avery Peterson, Evelyn Sebastian, Xiangkun Xu

**Affiliations:** ^1^ Department of Radiation Oncology William Beaumont University Hospital/ Corewell Health Royal Oak Michigan USA; ^2^ Department of Radiation Safety William Beaumont University Hospital/ Corewell Health Royal Oak Michigan USA

**Keywords:** brachytherapy, CT simulator, Gamma Knife, linacs, radiation shielding, radiation surveys, survey meters

## Abstract

**Background and purpose:**

Radiation shielding surveys require location and evaluation of radiation hotspots in shielding barriers. This process is normally done by using a conventional survey meter and slowly moving the survey meter manually across the face of the barrier. It would be advantageous to have a radiation image of the barrier showing hot spots. This would hasten the process, potentially reveal hidden hot spots and provide clear and unprecedented documentation for a variety of radiation sources, including HDR afterloaders, linacs, Gamma Knife and CT simulators. The purpose of this study is to evaluate a new survey instrument that can superimpose radiation “heat” maps on optical images, allowing the user to “see” the radiation distribution.

**Methods:**

A new survey instrument, the RAVIN (manufactured by M3D, Inc. Ann Arbor MI) has been evaluated for potential use in shielding surveys of linac vaults, HDR suites, Gamma Knife, CT simulators, and the search for lost radioactive seeds. This instrument also provides a NIST traceable calibrated measurement of the exposure rate or the air‐kerma rate. In addition, the instrument can show the spectrum of the measured radiation and can identify a wide range of commonly used isotopes. Comparisons have been made with conventional survey meters.

**Results:**

Heat maps have been acquired for the open door of a linac vault, linac head activation, an HDR afterloader, a Gamma Knife, the entrance to a CT simulator and an I‐125 seed. The camera provides some interesting information about the spectrum of radiation encountered in different shielding circumstances in radiation oncology. An example is the spectrum of radiation emerging from a thick linac 18 MV concrete primary barrier. The instrument shows a pronounced peak between 100 and 150 keV, with a long high energy, but low amplitude tail. Comparison with ionization chamber survey meters shows that this instrument can accurately measure air kerma rates from continuous high energy sources of radiation but cannot be used to measure air kerma rates from pulsed linac beams due to its relatively long dead time of 30 µs.

**Conclusion:**

The RAVIN camera is an innovative instrument that allows visualization of radiation under some circumstances while simultaneously accumulating an energy spectrum. The ability to “see” radiation is remarkable. This instrument is, however, probably not useful for routine radiation surveys of barriers in radiation oncology. This is due to the inability to reliably image heat maps for low levels of radiation (as are generally encountered outside shielding barriers) and to accurately localize the source.

## 1 INTRODUCTION

Radiation shielding surveys require location and evaluation of radiation hotspots in shielding barriers. This process is normally done by using a conventional survey meter and slowly moving the survey meter manually across the face of the barrier. It would be advantageous to have a radiation image of the barrier showing the hot spots. This would hasten the process, potentially reveal hidden hot spots and provide clear and unprecedented documentation for a variety of radiation sources, including HDR afterloaders, linacs, Gamma Knife units and CT simulators.

**FIGURE 1:**
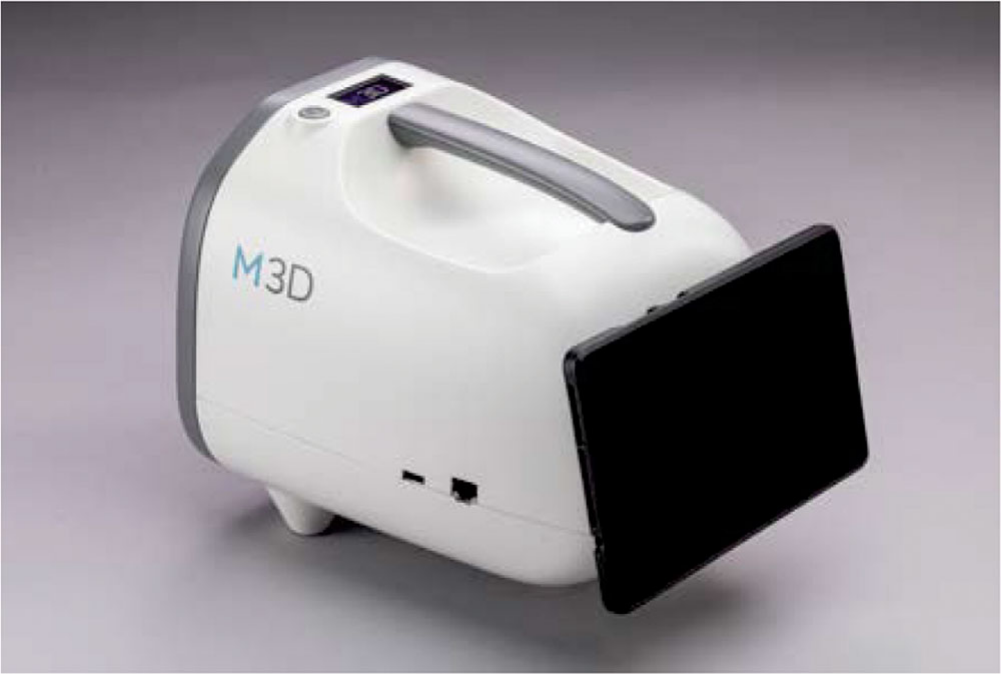
The M3D RAVIN imaging survey meter. A rear view of the meter showing the detachable imaging tablet. The imaging tablet communicates with the camera via Wi‐Fi and allows remote control of the instrument. A tripod can be used to position the instrument.

The M3D RAVIN (manufactured by M3D, Inc. Ann Arbor MI), is a new type of radiation survey instrument that permits visualization of a radiation “heat map” superimposed on an optical image.^1^ The instrument can measure exposure and air‐kerma rates. It also measures the spectrum of the radiation imaged. The RAVIN consists of two components, an imager and a detachable computer tablet which controls the device and displays images. The 8″ tablet communicates with the imager via Wi‐Fi. In addition, the RAVIN can connect to any smartphone via Wi‐Fi or to a laptop or desktop computer via ethernet cable. A photo of the RAVIN is shown in Figure 1. The RAVIN comes with an optional tripod. The tripod can be mounted on wheels for easy movement. The unit is battery powered. The battery life is 6+ hours, and it can be charged in 2 to 3 h from full discharge.

The M3D RAVIN camera contains a small gamma imaging spectrometer and a co‐registered stereoscopic optical camera. The field of view is approximately 90° horizontally and 60° vertically. An infrared sensor allows determination of distances to objects of interest (for alignment of the heat image with the optical image). The distance is needed to accurately back project the radiation heat map onto the optical image. If the distance is not correct this superposition may be faulty. There is a circular “bullseye” target superimposed on the optical image so the user can clearly see where the device is pointed. A lidar distance measuring device provides the distance. We have checked this against a tape measure and found it to be accurate in various circumstances.

The gamma spectrometer contains four cadmium‐zinc‐telluride (CZT) crystals arranged in a 2 by 2 array. The properties of CZT for radiation detection are described in standard references.^2^ For low energy photons (24 keV to 250 keV) the camera uses a “rank‐19 MURA coded aperture mask.” A back projection algorithm generates images of gamma emission. For photon energy above 250 keV, “both coded aperture and Compton images are produced.”

The gamma spectrometer measures a real‐time spectrum in the energy range from 25 keV to 665 keV for the standard RAVIN and from 25 keV to 1.2 MeV for the high energy RAVIN with energy resolution of 1% (FWHM) at 662 keV. The bin width is 1 keV. The high energy RAVIN was unavailable to us at the time measurements were made. The instrument generates a text file of the spectrum that can easily be imported into an EXCEL spreadsheet. The file contains the total number of counts in each of the energy bins. The time interval over which the counts were acquired is also listed. From the measured spectrum, the camera can identify a variety of commonly used isotopes based on a built‐in (user‐configurable) isotope library. The camera reconstructs images of the gamma emission and overlays these on an optical image. The company M3D reports that energy calibration is carried out in the range between 30 keV and 663 keV.^1^ Therefore, the reliability above 663 keV is uncertain. For linac measurements, the device should be in high energy scatter mode. At the bottom of the screen, it should say “high scat.” In this mode, the RAVIN forms the radiation hotspot image using the full spectrum of measured gammas/x‐rays from 30 to 663 keV.

The RAVIN will measure and display real time counts per second, instantaneous exposure rates in units of µR/h or air‐kerma rate in units of µSv/h. The manufacturer provided an optional NIST traceable Cs‐137 calibration certificate. When the count rate exceeds 30,000 cps, the detector will start to experience the effects of dead time, but the camera will still function and image. The reported air kerma rate will become less reliable. When the count rate exceeds 60,000 cps, the camera will become completely saturated and may stop working. It is then necessary to cycle the power to get it working again. The instrument cannot be used to measure exposure or air kerma rate from pulsed linac beams because of the relatively long dead time of 30 µs. However, we found that the instrument is in good agreement with calibrated ionization chamber survey meters for high energy continuous radiation sources.

The device can be placed in “time‐lapse” mode during which it will integrate for a set period of time (configured by the user) and then, after this elapsed time, will save the current image, clear the screen, and begin to form a new image. This allows the ability to trace a moving source (e.g. radioactive drug uptake during a radiopharmaceutical therapy treatment).

This instrument has been tested for applications with an HDR unit, a linac, a Gamma Knife and a CT simulator. In addition, tests were made with an I‐125 seed. The primary potential routine application of interest is the ability to show hotspots in shielding.

This instrument reveals some interesting information about the spectrum emerging from primary linac barriers. This radiation has a pronounced spectral peak at low energy, on the order of 150 keV (for 18 MV), with a long low intensity high energy tail. This is consistent with previous Monte Carlo (MC) calculations.

Several ionization chamber survey meters (Fluke 451P‐RYR) were used to make independent radiation level readings. In addition to this, a Fluke RaySafe 452 survey meter was also used. The RaySafe instrument combines an energy‐compensated Geiger‐Muller counter with diode detectors. All survey meters were calibrated within a year of the measurements. The RAVIN showed a background radiation level of 0.05 µSv/h.

## 2 APPLICATIONS TO AN IR‐192 HDR AFTERLOADER

Potential applications of the RAVIN to HDR are: 1) locating the position of a stuck source; 2) checking the integrity of the room shielding; and 3) surveying patients after treatment.

The RAVIN was tested for application with a Flexitron Ir‐192 afterloader. The source activity was 6.98 Ci on the day of testing.

The RAVIN was initially positioned at the front (the convex side opposite the handle) of the afterloader with the source in the safe at about 30 cm from the afterloader. The RAVIN showed an air‐kerma rate of 0.29 µSv/h. This was compared to the reading from two Fluke 451P‐RYR ionization survey meters at approximately the same distance. The ionization chambers were positioned at approximately the location of the front of the RAVIN. These survey meters showed air kerma rates of 0.30 µSv/h and 0.25 µSv/h. RAVIN did not show a heat map under these circumstances. The longest measurement with the RAVIN was taken for 161 seconds, which may not have been long enough to form a radiation image for such low air kerma rates.

**FIGURE 2:**
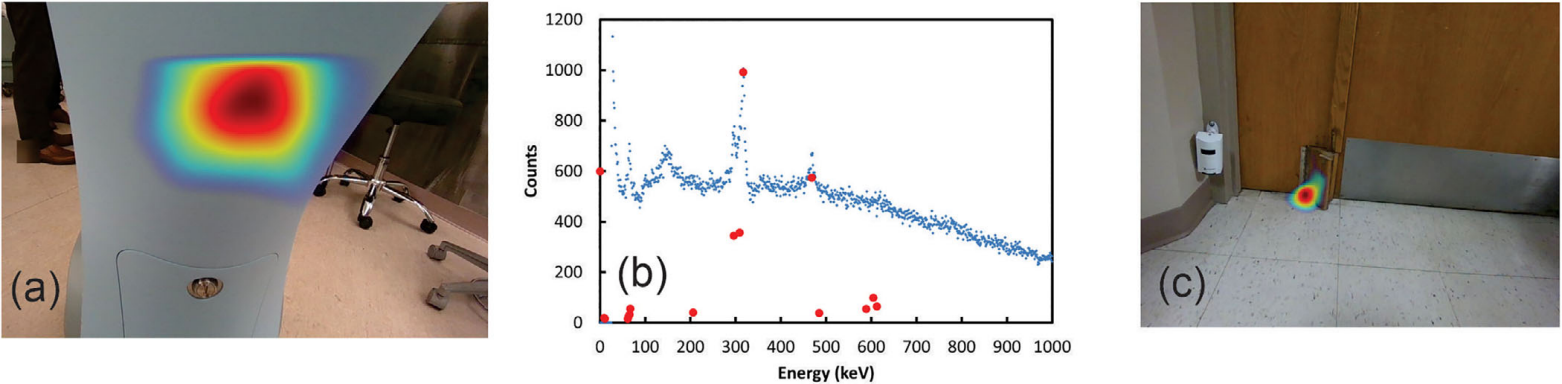
(a) HDR “heat” map. Shows the “heat” map acquired with the RAVIN at 30 cm from a Flexitron HDR afterloader containing an Ir‐192 source with an activity of 6.98 Ci. The air‐kerma rate at this location was about 0.50 µSv/h. (b) RAVIN spectrum of a “bare” Ir‐192 source. The red dots show the positions and relative amplitudes of known Ir‐192 spectral lines. RAVIN correctly identified this radioisotope. The Ir‐192 photopeak at 316 keV is prominent. (c) The heat map at the “mouse hole” in the HDR suite door.

The RAVIN was next located at the (concave) back of the afterloader, at about 30 cm from the afterloader. The reading of the RAVIN in this location was 0.47 µSv/h. Integration time was 286 seconds. The two ionization chamber readings were both 0.50 µSv/h. The RAVIN showed a heat map at the location of the source residing in the safe. This heat map is shown in Figure 2(a).

The RAVIN was positioned in the room while the source was sent out to reside in the check ruler for source positioning evaluation. The RAVIN correctly identified the isotope as Ir‐192. The RAVIN sometimes showed a heat map of the correct location of the source but at other times showed a ghost image where the source could not possibly reside. This may be due to incorrect distance measurements in a cluttered field of view. The spectrum displayed by RAVIN (Figure 2(b)) shows prominent Ir‐192 spectral lines at 67, 316 and 468 keV. The peak at 144 keV may be a backscatter peak associated with the 316 keV photopeak. The Compton edge associated with this peak is 175 keV and the backscatter peak is expected to have an energy of 316–175 = 141 keV.

The treatment room has a maze. The RAVIN was positioned outside the treatment room door at about 1.4 m from the door. This door has a small opening at the bottom (“mouse hole”) for cable passage. In the first set of measurements the mouse hole was closed. The source was then extended inside the room. The RAVIN showed an air kerma rate of 0.11 µSv/h and the ion chamber survey meters showed 0.09 µSv/h and 0.14 µSv/h. For an image integration time of 294 seconds, the RAVIN did not display a heat map of the door under these circumstances. The mouse hole was then opened, and the readings were repeated. The distance was reduced to 1.1 m. Under these circumstances it was possible to acquire a hotspot image at the location of the mouse hole (see Figure 2(c)). The RAVIN showed an air kerma rate of 1.58 µSv/h, whereas the ion chamber survey meters showed readings of 2.20 µSv/h and 1.00 µSv/h.

The size and weight of the RAVIN make it somewhat unwieldy for patient surveys. The RAVIN camera weighs 5 lbs, in contrast to the Fluke 451P‐RYR, which weighs 2.4 lbs. It is also bulkier. The flat energy response and the size and weight of the ionization chamber survey meter favor that device for patient surveys.

As for locating a stuck source, the RAVIN response is not rapid enough and the appearance of a heat map not reliable enough for this purpose. The RAVIN exposure rate measurements appear to be in good agreement with ion chamber measurements. We found the generation of heat maps to be somewhat temperamental. It seems that heat maps are only generated when the exposure rate is between certain limits.

## 3 APPLICATIONS TO LINACS

Potential applications for linacs are locating hotspots around barriers (both primary and secondary), doors and room penetrations. Another potential application is evaluation of head leakage. For linac measurements the device should be in the “Scatter High” mode. This images a broad range of scatter in the 30–600 keV range.

### 3.1 Door

**FIGURE 3:**
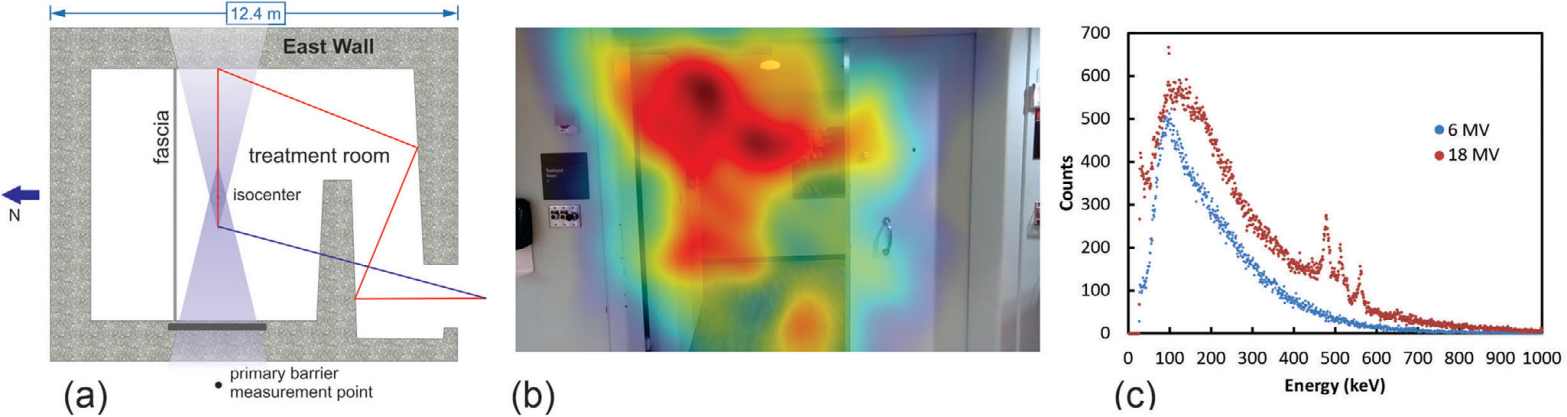
(a) Plan view of the linac vault surveyed with the RAVIN. The red pathway shows a nominal primary photon path to the door when the beam is pointed at the east wall (top). The photon must scatter three times to reach the RAVIN at the doorway. The blue path shows head leakage photons reaching the door after passing through the maze wall. Calculations suggest that the dominant component of the radiation field outside the door is due to head leakage transmitted through the maze wall. The west wall primary barrier is composite, consisting of concrete and lead. (b) The heat map for the 6 MV beam with the linac vault door open. The beam is pointed at the east wall. The hotspots are likely due to a combination of leakage radiation transmitted through the maze wall and primary radiation scattered down the maze from the east wall. (c) The spectra at the open door of the treatment room for 6 MV (blue) and 18 MV (red). The relative amplitudes are not meaningful. The beam was pointed at the east wall. The 18 MV spectrum shows sub‐peaks at energies of 479 keV and 562 keV. The 511 keV line is presumed to be due to positron annihilation.

We were unable to image any hotspots at the door with this instrument, even though it was set to high energy mode, except in the case where the door interlock was bypassed and the door was open. With the door closed, the “dose” rate was only about twice background (the background level was 0.05 µSv/h), and this level may be too low to obtain a heat image (for both 6 MV and 18 MV). The fact that we couldn't acquire a heat map around the closed door may be due to the design of this vault (see Figure 3(a)). Photons must scatter three times to exit the door, or they must pass through the maze wall.

The heat map obtained with the door open and the 18 MV beam on showed an anomaly—a hot spot where there is shielding. We believe this is because the side of the door is at a significantly different distance than the door itself. The geometry of this room is such that the plane of the door is parallel with the maze centerline (see Figure 3(a)). This is described as a three‐leg maze in IPEM Report 75; “Design and Shielding of Radiotherapy Treatment Facilities.”^3^ NCRP151 does not consider the case of a three‐leg maze^4^. The gantry was pointed at the east wall (gantry angle 270°). The collimator angle was 45° and the field size was 35 × 35 cm^2^. The linac “dose rate” for 6 MV was 510 MU/min and for 18 MV it was 554 MU/min. The distance from the inner maze wall, as seen looking through the open door, was 4.5 m. Figure 3(b) shows the heat map for the 6 MV beam with the door open. Figure 3(c) shows the spectra for 6 MV and 18 MV under these circumstances.

When the beam is pointed at the east wall there are three contributions to the radiation level at the door: 1) radiation reflected off the east wall (red pathway in Figure 3(a)), 2) head leakage scattered off the walls and 3) head leakage transmitted through the maze (blue pathway). Shielding calculations have been performed following IPEM Report 75 to evaluate the various components of the radiation expected to reach a point just outside the door with the door open and the gantry pointed toward the east wall. Instantaneous radiation levels have been estimated based on the instantaneous dose rate at isocenter. Patient scatter has been ignored because no phantom was in the beam. For 6 MV the instantaneous air kerma rate is expected to be 2.1 µSv/h and 11 µSv/h for 18 MV. For both 6 MV and 18 MV, the head leakage transmitted through the maze wall dominates the other contributions.

For the spectra shown in Figure 3(c), the relative amplitudes are not meaningful as the dose rates and the acquisition times for the two beams are different. The 6 MV spectrum peaks at about 100 keV and the 18 MV spectrum peaks at around 125 keV. The 18 MV spectrum also shows minor peaks at 479, 512 and 562 keV. The 512 keV peak is presumed due to pair production in this high energy beam. The peak at 479 is believed to be due to boron neutron capture (known energy 478 keV) and the 560 keV line to cadmium neutron capture (known energy 558 keV). These lines are believed to originate within the instrument itself as a result of neutron irradiation. ^5^

When the gantry is pointed at the east wall, primary photons must undergo three scattering events to reach the instrument. A typical set of scattering angles is shown in Figure 3(a). These angles are 112°, 91° and 113°. A 6 MV linac beam has an average energy of about 2 MeV. Such photons scattered through the angles above would have an energy of 127 keV. For 18 MV, the average photon energy is about 5 MeV.^6^ The scattered photons in this case would have an energy of about 131 keV.

### 3.2 Primary barrier

We were unable to acquire a “heat” map for the primary barrier at the location labeled “primary barrier measurement point” shown in Figure 3(a). The irradiation conditions were: gantry pointed toward barrier, 18 MV, collimator angle 45°, field size set to 35 cm by 35 cm, “dose” rate = 532 MU/min. The barrier is approximately 1.2 m thick. It is a composite barrier containing standard density concrete and lead. The RAVIN showed a “dose” rate of 2.2 µSv/h. The ionization chambers showed 11 µSv/h and 8.7 µSv/h. This is approximately a factor of 5 higher.

The anomalously low RAVIN reading may be due to the pulsed nature of linac radiation. The linac delivers radiation in pulses with a duration on the order of 5 µs. Pulses are repeated about every 5 ms. The RAVIN instrument has a relatively long dead time of about 30 µs.^5^ This is about six times longer than the duration of an individual linac pulse. It is therefore not possible to measure radiation exposure or “dose” rates around medical linacs with this instrument. Measurement of radiation levels is probably best accomplished with an ionization chamber survey meter rather than with a solid‐state detector which can saturate, and which has a significant energy dependence.

**FIGURE 4:**
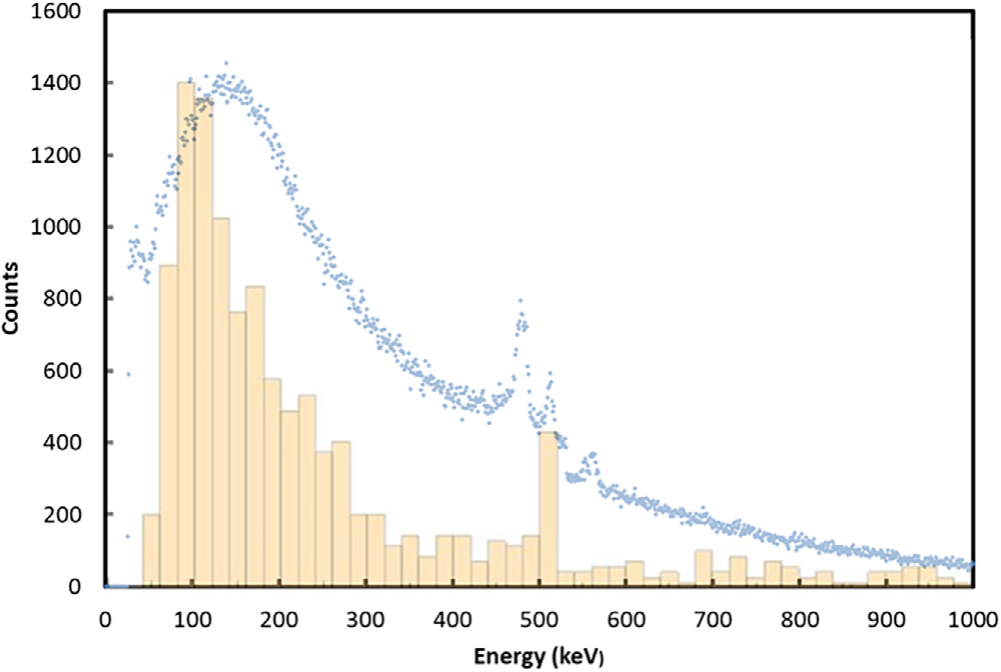
RAVIN spectrum (blue) superimposed on MC calculated spectrum (yellow) for 18 MV radiation traversing a 2 m thick concrete primary barrier. The vertical axis shows counts per bin. The RAVIN bin width is 1 keV. Both spectra show a peak near 100 keV and an annihilation line at 510 keV. The RAVIN spectrum also shows peaks at 480 keV and 560 keV. These are believed to be neutron capture lines originating in the camera.

A spectrum was acquired for the primary barrier of this linac under the conditions described above. The statistical noise was reduced by summing about a half dozen different acquisitions (see Figure 4). This spectrum shows a pronounced peak at about 140 keV, with smaller peaks at 480 keV, 510 keV and 560 keV. The 510 keV peak is presumed to be due to pair production and subsequent electron/positron annihilation. The 480 keV line is a boron neutron capture line (478 keV) and the 560 keV line is a cadmium neutron capture line (558 keV). Both lines are thought to originate within the camera itself.^4^

For comparison purposes, a MC computed spectrum for the emergent radiation for an 18 MV beam traversing a 2.0 m thick concrete (no steel or lead) barrier has been superimposed on the measured spectrum. The MC calculations have been described in the paper by McDermott.^7^ These calculations do not model the instrument response and do not include any interactions that take place within the instrument such as Compton scattering, activation, etc. The observed spectrum is qualitatively consistent with the results of the MC calculations. The MC spectrum also shows a 511 keV annihilation line. The MC spectrum shows a peak at an energy of about 90 keV with a very long, high energy but low amplitude tail. The mean energy of the MC emergent radiation from a 2.0 m concrete barrier with a distal distance of 5 m is about 2.2 MeV. The beam is not hardened, as commonly thought, but rather dramatically softened because very few primary photons are able to penetrate the barrier and the vast majority of photons emerging have been multiply scattered.

### 3.3 Head leakage

At one time, it was common to assess head leakage by covering the linac head with ready‐pack film. It would be very convenient to image the head leakage directly. This might be accomplished by imaging the head from the sides, the front and the top. The RAVIN was placed on a tripod at various distances from the linac head. The linac was operated at 6 MV at a gantry angle of 90° with no material in the beam. Our attempt to produce a heat image failed. The count rate exceeded 60,000 cps and the camera became completely saturated.

### 3.4 Head activation

**FIGURE 5:**
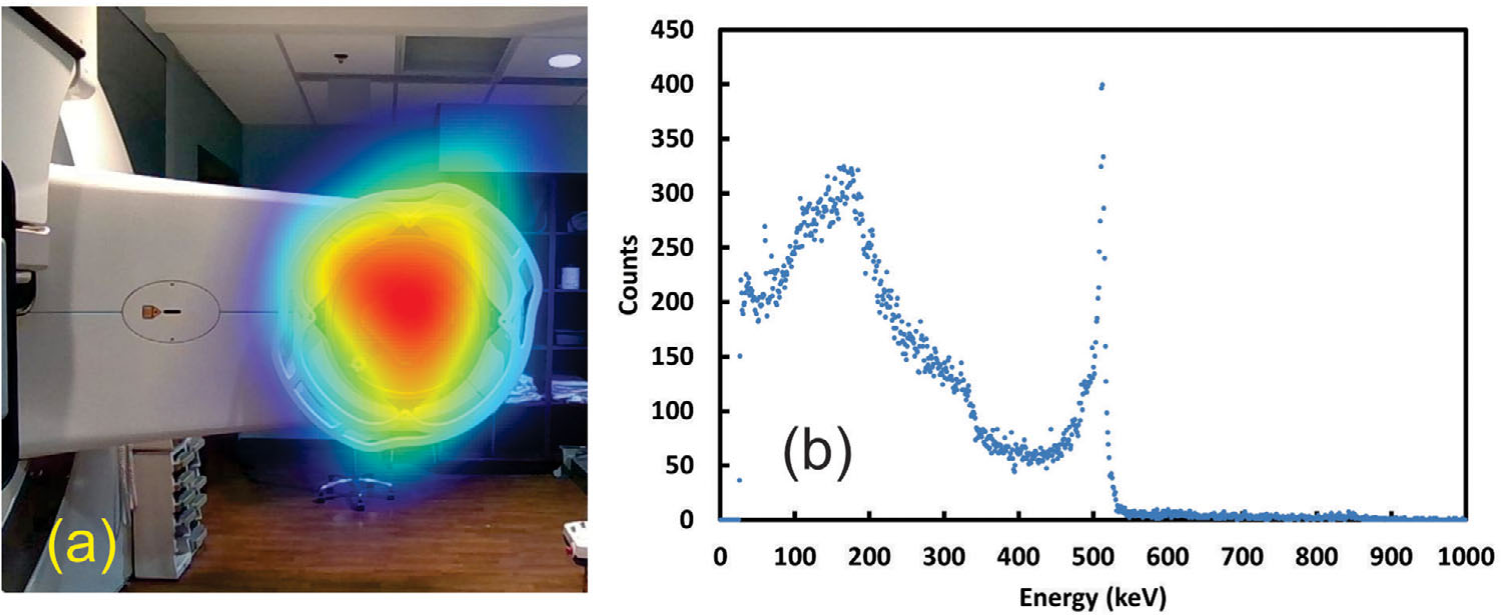
(a) “Heat” image showing linac head activation after prolonged operation at 18 MV. This image was acquired over a 34 s time interval. (b) The activation spectrum from the linac head shows a very pronounced annihilation peak at 511 keV and very few counts at energies higher than this. The broad peak at 170 keV is likely the backscatter peak from the annihilation radiation.

The Elekta linac head was imaged after running 18 MV in open loop for a lengthy period of time (on the order of 15 minutes). An activation image was created with the gantry at 90° and the camera pointed at the head (see Figure 5(a)) at a distance of about 2 m. The acquisition time was 34 s. The RAVIN measured air kerma rate at this distance was 1.3 µSv/h. The spectrum is shown in Figure 5(b). The spectrum displays a broad peak at about 170 keV and a very sharp peak at 510 keV. The amplitude drops sharply for energies greater than 510 keV. The calculated Compton edge associated with the annihilation peak has an energy of 340 keV and the calculated backscatter peak has an energy of 170 keV. This spectrum is qualitatively similar to the spectrum shown in the paper by Fischer, Tabot and Poppe, although the broad peak at 170 keV in our spectrum has a higher amplitude than theirs.^8^

## 4 GAMMA KNIFE

RAVIN measurements were made for an Elekta Leksell Gamma Knife^®^ ICON™ unit. The dose rate at the center of a 7.5 cm radius sphere of solid water at the unit center was 2.92 Gy/min. It is estimated that the total activity of the sources at the time of measurement was 4122 Ci. (1.4 × 10^14^ Bq) on 12/8/2025.

**FIGURE 6:**
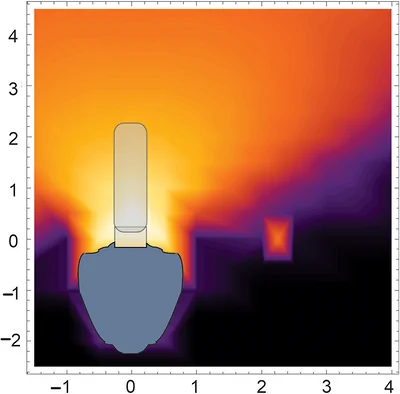
A plan view color wash of relative radiation levels from the Gamma Knife on a plane 1 m above the floor with a 16 mm collimator and open shield doors. Distance labels are in meters. Radiation levels are highest along the long axis of the couch and at small angles to this axis.

Figure 6 shows a color wash map of the radiation levels for the ICON on a plane 1 m above the floor with the 16 mm collimator and open shielding doors. The data to construct this map were taken from the *Leksell Gamma Knife Icon: Site Planning Guide*.^9^ It is not clear, based on this reference, whether a phantom was in the treatment position. Figure 6 was constructed by taking the logarithm of the quoted radiation levels in µSv/h for various grid positions. Absolute radiation levels will depend on the activity of the Co‐60 sources.

**FIGURE 7:**
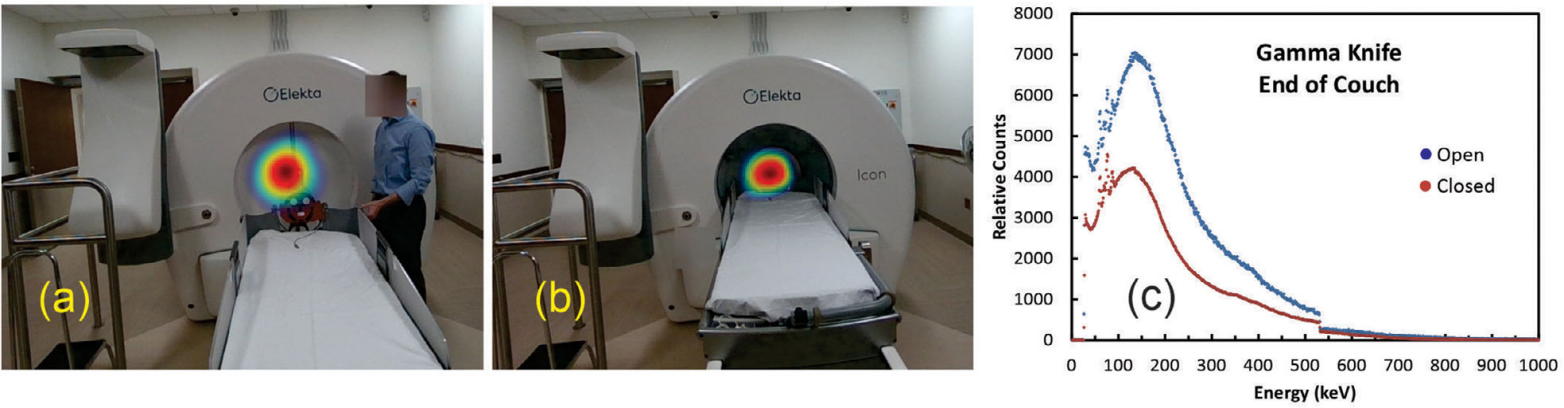
The heat map imaged at the end of the treatment couch at 2.9 m from the outer surface of the shielding doors with doors closed (a) and doors open (b). The predominant contribution to the radiation when the doors are closed is through the doors. The image with the doors open was made with a 16 mm collimator setting. (c) Relative counts vs energy for the Gamma Knife at the foot of the couch with the shielding doors closed (red) and with the doors open (blue). For doors open the 16 mm collimator was used and there was a CATPHAN phantom at the unit center. The relative amplitudes of the two spectra are meaningless as the acquisition time was different for the two spectra.

The first set of measurements were made at the end of the treatment couch at 2.9 m from the shielding doors with the doors closed. The RAVIN measured a radiation level was 0.27 µSv/h. The Fluke 451P‐RYR instrument measured 0.30 µSv/h. The heat map is shown in Figure 7(a). All the radiation shown on the heat map is coming through the shielding doors.

The spectrum in Figure 7(c) shows a broad peak at 120 keV and a sharp peak at about 76 keV. It is speculated that the latter feature may be the Kα1 line from lead which has a known energy of 75 keV. This might be expected from photoelectric interactions if the shielding doors contain lead. The average energy is 204 keV. There are very few photons with energy exceeding 550 keV. There is a sharp drop off at an energy of about 530 keV. The origin of this is unknown.

These measurements were repeated with the doors open without moving the camera. The 16 mm collimator setting was used with all channels open. A CATPHAN CT phantom was positioned at the unit center to provide scatter to mimic a patient. The CATPHAN (model 503) is a 20 cm diameter cast epoxy cylinder. With the doors, open the RAVIN measured 53 µSv/h. The shielding data provided by Elekta predicts an air kerma rate of about 240 µSv/h. This is presumed to be in the absence of a phantom at the unit center and it is probably for fresh Co‐60 sources. This is a high gradient location.

The spectrum is shown in Figure 7(c). There is a broad peak at 130 keV and the average energy is 204 keV. Photons with an energy of 1.25 MeV (average for Co‐60) scattered through an angle of 90° are expected to have an energy of 360 keV. There is perhaps a hint of a slight bump in the open‐door spectrum at that energy, but it is certainly not pronounced.

IPEM Report 75 presents a spectrum for the Perfexion obtained from Elekta “at wall 3m from RU.” It is not clear what “3m from the RU” means.^1^ This spectrum does not show any primary radiation at 1.17 MeV or 1.33 MeV. This spectrum shows a sharp peak at about 120 keV and a broader peak at about 250 keV. The design of the Perfexion is such that none of the Co‐60 beams exit through the shielding doors. Radiation exiting the unit is apparently overwhelmingly scatter. The low average energy of the emitted spectrum in both the open and closed state show that the assumption of Co‐60 energy radiation in shielding calculations is extremely conservative and will result in a very heavily shielded treatment room.^10^ According to IPEM 75, Elekta plans to derive “effective tenth value layer” values. In the absence of this data, the typical approach to shielding is to assume primary Co‐60 radiation. TVL data for the scattered radiation would likely significantly reduce the shielding requirements.

Matias et al (2023) have made HPGe detector measurements and MC calculations of the spectrum of a GK Perfexion.^11^ These authors measured the spectrum at about the same position as reported above. Their spectrum is in good agreement with that shown in Figure 7(c) up to an energy of 350 keV. Beyond that energy, the Matias spectrum is much higher in amplitude Their spectra extend up to a maximum energy of 2 MeV. They too, show a peak in the open‐door spectrum (with a phantom in the unit, 16 mm collimator) at about 130 keV. They also show a rapid drop off in counts above about 630 keV. Their MC calculations show that the scatter contribution from the floors and walls is significant.

**FIGURE 8:**
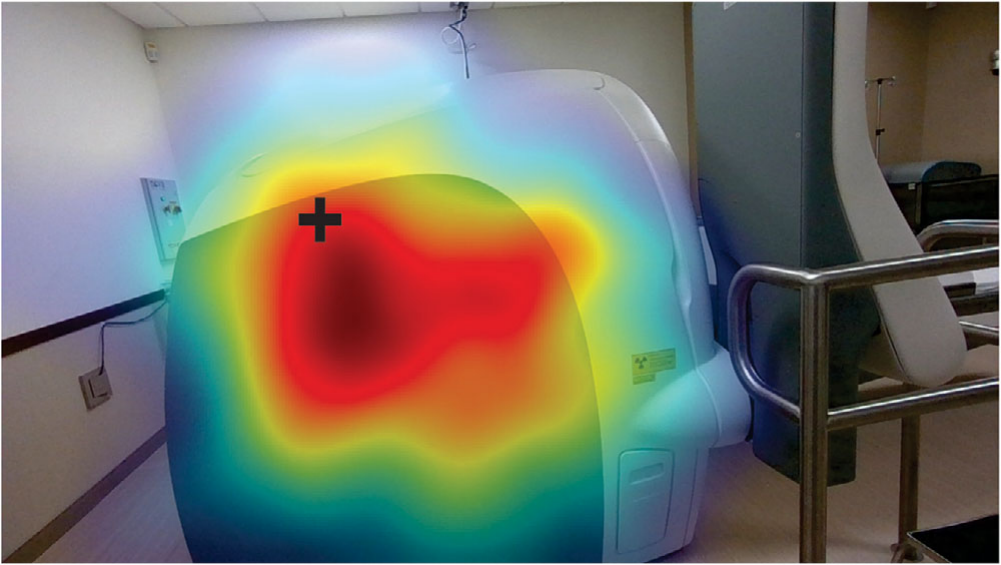
Heat map of the side of the Gamma Knife at 1.6 m from the surface of the unit. Shielding doors are closed. The black cross indicates the position of the hotspot as determined with an ionization chamber survey meter.

Measurements were made at the side of the Gamma Knife at approximate coordinates *x* = 2.5, *y* = −1 in Figure 6. The heat map is shown in Figure 8. The distance from the surface of the Gamma Knife housing is 1.6 m. The RAVIN radiation level was recorded as 0.44 µSv/h. The Fluke 451P‐RYR ionization chamber read 0.45 µSv/h. The location of the hotspot did move around to some degree from exposure to exposure. The black cross displayed in this figure shows the location of the hotspot as determined with an ionization chamber survey meter. This determination was made independently without looking at the heat map.

An unsuccessful attempt was made to acquire a heat map at the closed treatment room door with the shielding doors open and the collimator set to 16 mm. The Fluke 451P‐RYR survey meters showed radiation levels of about 0.11 µSv/h (readings fluctuated from 0.05 µSv/h to 0.15 µSv/h) at 2 m from the door. The measured Fluke 451P‐RYR radiation level was not noticeably different with the shielding doors closed. The RAVIN showed a radiation level of 0.05 µSv/h. The failure to acquire a heat map is likely because radiation levels are almost at background level at this location.

## 5 IODINE‐125 SEED

**FIGURE 9:**
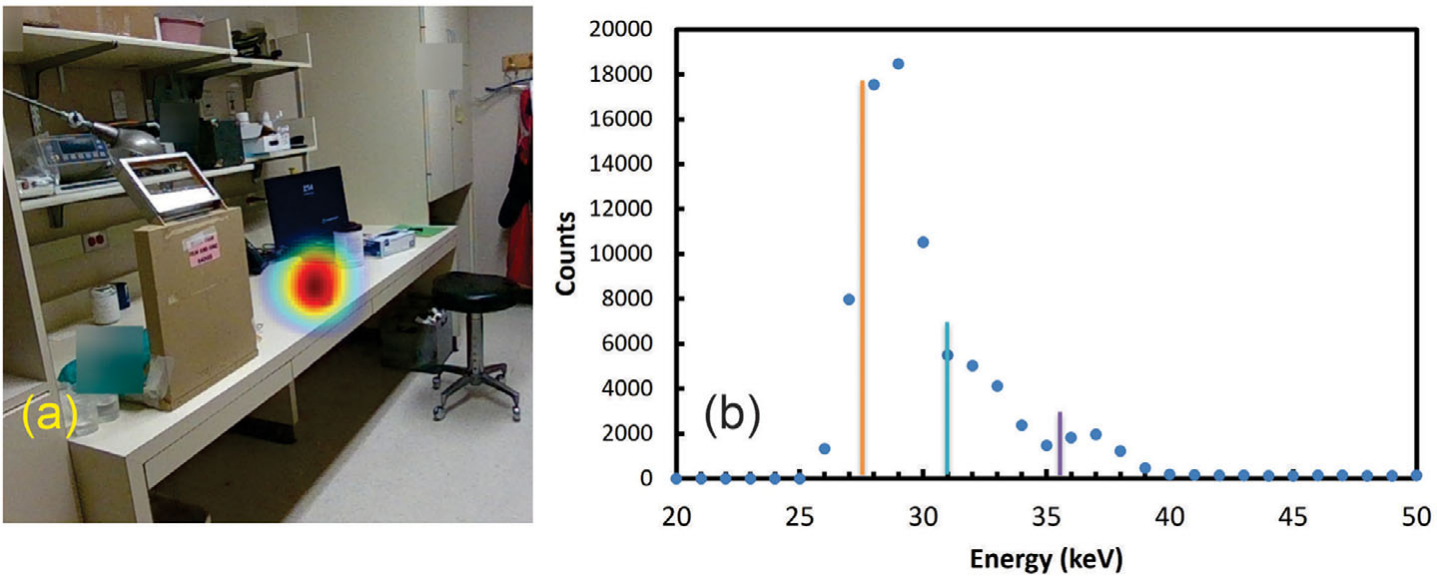
(a) Heat image of a 2.6 mCi I‐125 source at 2 m. This image accurately depicts the location of the source. (b) The spectrum of the 2.6 mCi I‐125 seed (Theragenics AgX 100). The vertical lines indicate the energies and relative amplitudes of known spectral lines.

The RAVIN camera has been tested by attempting to image a Theragenics AgX100 I‐125 seed. The lowest strength seed available to us had an activity of 2.57 mCi (used for eye plaques) on the date of measurement. I‐125 seeds used for prostate implants have much lower activity, typically on the order of 0.4 mCi. The seed was removed from its shielded container and placed on the counter of the hot lab. The RAVIN was mounted on a tripod outside the hot lab room and positioned in the doorway at 2.0 m from the seed. The source showed a clear heat image at the correct location as seen in Figure 9. If this source was lost, RAVIN would allow a rapid and accurate determination of its location, provided that the distance is correct. If the distance is incorrect there could be an error in the displayed position.

The radiation level measured by RAVIN was 0.09 µSv/h. The Fluke 450P‐RYR instruments measured 0.50 µSv/h and 0.57 µSv/h. The Fluke RaySafe 452 measured 0.87 µSv/h. Gamma ray constants for I‐125 seeds range from Γ = 1.35 R cm^2^ mCi^−1^ h^−1^ to 1.45 R cm^2^ mCi^−1 ^h^−1^. The radiation level calculated based on these values is 0.88 µSv/h to 0.95 µSv/hr. For the 450P‐RYR, the energy response drops to about 0.5 at 30 keV compared to the response at the calibration energy of Cs‐137 (660 keV) for measurements when the radiation enters the front of the instrument.

Figure 9(b) shows the spectrum measured by the RAVIN. Known spectral lines for I‐125 have energies of 27.4 keV, 31.0 keV and 35.5 keV. The figure shows vertical marks on the horizontal axis at these known energies and approximate amplitudes (normalized to the 27 keV line). The manufacturer (M3D) indicates the RAVIN has not been calibrated at these low energies.

## 6 CT SIMULATOR

**FIGURE 10:**
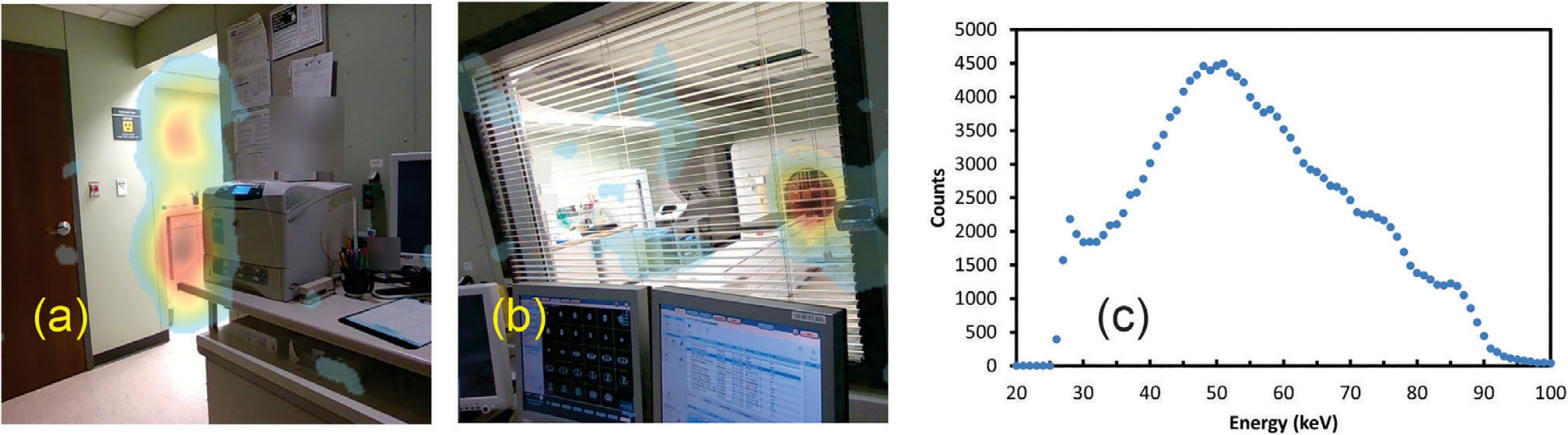
Heat map for entrance to CT simulator room from the control room (a) and heat map at the leaded glass window of the control room (b). Hotspots show radiation scattered from the wall facing the entrance. Radiation emerging from the leaded glass window is coming from the bore of the CT. (c) The spectrum of radiation emerging from the open door of the control room of the CT simulator. The CT was scanning a phantom at 120 kVp.

The RAVIN camera was used to survey a Philips Brilliance Big Bore CT simulator. A RANDO phantom was scanned with a low pitch of 0.56 to keep the CT tube energized for long enough to obtain measurements and to provide scatter. The CT settings were 120 kVp and 320 mAs. The camera was set up to image the open entryway from the control room to the CT room. An image was obtained (see Figure 10(a)) showing radiation coming through the doorway that has been scattered from the wall visible from the doorway.

The peak of the spectrum shown in Figure 10(c) is at about 50 keV. According to Parrott, et al., the average energy of 120 kVp CT x‐ray beam is 67 keV.^12^ Compton scattering at these energies results in scattered photons that lose very little energy in the scattering process: $E_{\gamma^{\prime }}\approx 0.9E_{\gamma }$ for 90° scattering. This is almost at the Thomson scattering limit.

## 7 CONCLUSION

The RAVIN camera is an innovative instrument that allows visualization of radiation under some circumstances while simultaneously accumulating an energy spectrum. The ability to “see” radiation is remarkable. This instrument is, however, probably not useful for routine radiation surveys of barriers in radiation oncology. This is due to the inability to reliably image heat maps for low levels of radiation (as are generally encountered outside shielding barriers) and to accurately localize the source. The linac that we surveyed is very heavily shielded. If there was a serious breach in the shielding, the instrument might show that. It would be very useful if the instrument was capable of routine imaging of low‐level hotspots. In addition, the instrument cannot be used to measure exposure or air kerma rates from pulsed linac beams due to its relatively long dead time of 30 µs. In discussions with M3D, they have indicated accurate imaging of pulsed beams is an area of ongoing research and development for the RAVIN camera.

The camera does provide some interesting information about the spectrum of radiation encountered in different shielding circumstances in radiation oncology. An example is the spectrum of radiation emerging from a thick 18 MV linac concrete primary barrier. The measurements suggest that the beam is significantly softened after passing through the primary barrier. The instrument shows a pronounced peak between 100 and 150 keV, with a long high energy but low amplitude tail. This is consistent with MC calculations that show that the beam is considerably softened when traversing a thick barrier. This is due to fact that the vast majority of photons emerging from the barrier have been scattered multiple times. Very few primary photons are able to penetrate the barrier and thus the average energy of the emerging photons is reduced significantly.

IPEM Report 75 states that “In practice the x‐ray beam will be hardened as it penetrates the shielding and the TVL will increase, especially after the first TVL.” This statement is not correct for a concrete barrier and is belied by the data in this very reference that shows TVL_e_ < TVL_1_ where TVL_1_ is the first TVL and TVL_e_ is the subsequent equilibrium TVL. If the mean free path of the highest energy component of the beam spectrum is much smaller than the barrier thickness, high energy photons will be multiply scattered and the emergent spectrum will be softened, not hardened.

The RAVIN does not always show the correct location of the HDR Ir‐192 source. This may be due to an incorrect distance to the source. The RAVIN may assist in locating a lost source but it will not always show a heat map, and when it does it is imperative that the distance be correct to get a heat map that accurately identifies the source location.

One of the questions that we have endeavored to answer is: can the RAVIN be used to locate lost I‐125 seeds? We have not been able to definitively answer this. The 2.5 mCi seed that we used had an activity that was about a factor five times larger than the seeds typically used for prostate implants. We had no trouble accurately imaging the location of this seed at a distance of 2 m. It is not clear whether a lower activity seed would be so easily imaged. An accurate image also depends on whether RAVIN has an accurate distance. If the distance is inaccurate, as likely with the case of a lost seed, RAVIN may show an inaccurate seed location.

Radiation levels measured for the Ir‐192 and Gamma Knife Co‐60 scatter radiation agree well with ionization chamber survey meter readings. However, the RAVIN does not come calibrated to provide accurate radiation level readings for low energy radiation such as that originating from I‐125 or a CT simulator and therefore would require further onsite calibration by the user for use in these applications for air kerma rate measurements.^2^ It is noted that ionization chamber survey meters calibrated for Cs‐137 are also inaccurate under these circumstances.

## AUTHOR CONTRIBUTIONS

**Patrick McDermott**: Participated in all measurements, data analysis and wrote the manuscript. **Robert Halford**: Participated in Gamma Knife measurements. **Brayden Halvorson**: Participated in all measurements. **Ian Lake**: Introduced the instrument and participated in some measurements. **Jian Liang**: Participated in CT simulator measurements. **Akin Ogretici**: Introduced the instrument and participated in some measurements. **Avery Peterson**: Participated in all measurements. **Evelyn Sebastian**: Participated in HDR measurements. **Xiangkun Xi**: Participated in all measurements.

## CONFLICT OF INTEREST STATEMENT

The authors have no relevant conflicts of interest to disclose

## ETHICS STATEMENT

The authors of this manuscript have no financial interest in the company M3D or in the RAVIN camera. The RAVIN camera was loaned to the authors for evaluation.

## FUNDING STATEMENT

There was no external funding for this research.

## Data Availability

There is no significant data available related to this study beyond that contained in this manuscript.

## Footnotes

1 The ICON is the same as the Perfexion except that it has cone beam CT capability.

2 This is a known issue that M3D is working to correct for version 2.0 of the RAVIN.

## References

[1] Sawyer S , Brown S , Polf J , Viglianti B , Characterization and dose rate calibration of a semiconductor‐based imaging survey meter. J Nucl Med 2025;66(supplement 1): 251852.

[2] Cherry SR , Sorenson JA , Phelps ME , Physics in Nuclear Medicine, 3rd ed. Saunders: 2003.

[3] Horton P and Eaton D , eds. Design and Shielding of Radiotherapy Treatment Facilities, IPEM Report 75, 2nd ed. IOP Publishing: 2017.

[4] NCRP . Structural Shielding Design and Evaluation for Megavoltage X‐ and Gamma Ray Radiotherapy Facilities, NCRP Report No. 151. NCRP: 2005. 10.1118/1.2336250 28525064

[5] Polf J , M3D company, private communication: 2025.

[6] Shiekh‐Bagheri D , Rogers DWO , Monte Carlo calculation of nine megavoltage photon beam spectra using the BEAM code. Med Phys 2002;29: 391‐402. 10.1118/1.1445413 11930914

[7] McDermott PN , Linac primary barrier transmission for concrete: Monte Carlo calculations. J Appl Clin Med Phys. 2022;24(1): e13847. 10.1002/acm2.13847 PMC9859993 36471480

[8] Fischer HW , Tabot BE , Poppe B , Activation processes in a medical linear accelerator and spatial distribution of activation products. Phys Med Biol 2006;51(24): N461‐N466. 10.1088/0031-9155/51/24/N02 17148816

[9] Elekta . Leksell Gamma Knife Icon: Site Planning Guide, Elekta Instrument AB: 2016

[10] McDermott PN , Radiation shielding for gamma stereotactic radiosurgery units. J Appl Clin Med Phys. 2007;3(8): p147‐157. 10.1120/jacmp.v8i3.2355 PMC5722598 17712298

[11] Matias LDS , Benmakhlouf H , Doncel M , et al. Characterization of the radiation field surrounding the Lexsell Gamma Knife and shielding applications. Appl Radiat Isot 2023;198: 110839. 10.1016/j.apradiso.2023.110839 37244206

[12] Parrott D , Smereka P , Dane B , Dual‐energy CT systems: technical aspects and selected clinical applications. Br J Radiol 2025;1175(98): 1724‐1735. 10.1093/bjr/tqaf086 40249100

